# An investigation of clinical and immunological events following repeated aerodigestive tract challenge infections with live *Mycobacterium bovis* Bacille Calmette Guérin

**DOI:** 10.1016/j.vaccine.2010.06.005

**Published:** 2010-07-26

**Authors:** Fernanda Schreiber, Zhiming Huo, Rafaela Giemza, Maria Woodrow, Nicola Fenner, Zoe Stevens, Gordon Dougan, Steven Prideaux, Luiz R.R. Castello-Branco, David J.M. Lewis

**Affiliations:** a The Wellcome Trust Sanger Institute, The Wellcome Trust Genome Campus, Hinxton, Cambridgeshire CB10 1SA, United Kingdom; b St George's Vaccine Institute, St George's University of London, Cranmer Terrace, SW17 0RE London, United Kingdom; c Instituto Oswaldo Cruz and Instituto Nacional de Ciência e Tecnologia em Tuberculose, Av. Brasil 4365, CEP 21045-900 Rio de Janeiro, Brazil

**Keywords:** BCG, Oral challenge, Innate immunity

## Abstract

Bacille Calmette Guérin substrain Moreau Rio de Janeiro is an attenuated strain of *Mycobacterium bovis* that has been used extensively as an oral tuberculosis vaccine. We assessed its potential as a challenge model to study clinical and immunological events following repeated mycobacterial gut infection. Seven individuals received three oral challenges with approximately 10^7^ viable bacilli. Clinical symptoms, T-cell responses and gene expression patterns in peripheral blood were monitored. Clinical symptoms were relatively mild and declined following each oral challenge. Delayed T-cell responses were observed, and limited differential gene expression detected by microarrays. Oral challenge with BCG Moreau Rio de Janeiro vaccine was immunogenic in healthy volunteers, limiting its potential to explore clinical innate immune responses, but with low reactogenicity.

## 1 Introduction

*Mycobacterium bovis* based Bacille Calmette Guérin (BCG) was originally introduced in the 1930s as an oral vaccine against the human pathogen *Mycobacterium tuberculosis*, the cause of tuberculosis. In the 1960s, most of the world moved towards intradermal vaccination with lyophilized BCG, but some countries, including Brazil, continued to exploit the oral vaccination route [1,2]. BCG, which is still available as a live vaccine, was derived by extensive passage from *M. bovis*, which naturally infects humans and cattle via the gastrointestinal tract. Live *Mycobacteria* have the potential to interact strongly with both the innate and adaptive immune system and any vaccine based on them has the potential to be used as a safe clinical probe of human responses [3]. Thus, BCG-based vaccines can potentially provide a safe but effective tool to mimic natural infection and stimulate both innate and acquired immunity under relatively ‘natural’ conditions of gut infection. Further, as BCG is a licensed vaccine many ethical hurdles are consequently reduced for human studies.

Immune responses can be both protective and dangerous to the host. For example, many of the symptoms associated with the reactogenicity of vaccines are in fact inappropriately stimulated innate responses. Innate immune responses are difficult to safely monitor in humans as approved methods for stimulating such responses are not generally available and would raise ethical concerns. By delivering oral BCG (which has been given orally to millions of people with a good record of safety) to healthy volunteers under controlled conditions we aimed to assess if this system had value for monitoring innate immune activation. The impact of gastrointestinal colonization by BCG was indirectly determined by measuring antigen-specific T-cell and cytokine responses, along with microarray analysis. Further insight was obtained by systematically recording clinical symptoms associated with sequential BCG challenges such as abdominal pain, diarrhoea; upper respiratory tract congestion, secretion; fever and headache. In this way, we sought to build-up an integrated picture of innate and adaptive immune responses at various time points before and after a series of bacterial challenges. We used an oral BCG preparation (BCG Moreau Rio de Janeiro), commercially produced, which has a strong safety record in extensive human testing [4]. We report here data for seven subjects recruited into a pilot study to determine the feasibility of this approach, and to develop and test assays and protocols to identify innate immune pathways associated with gastrointestinal infections in humans.

## 2 Materials and methods

### 2.1 Subjects and ethics statement

Healthy volunteers were recruited to the study sponsored by St George's University of London, approved by St George's Research Ethics Committee (reference 06/Q0803/61). Prior formal review by the UK Competent Authority for regulating clinical trials, the Medicines and Healthcare products Regulatory Agency (MHRA), confirmed that this basic science challenge study was not a clinical trial as defined by UK and European Union legislation. To maximize subject safety the study was conducted in compliance with principles of Good Clinical Practice. The study is registered on ClinicalTrials.gov (NCT01074775).

Subjects were considered eligible for challenge if they were 18–45 years of age, in good health as determined by medical history and physical examination, had no clinically significant abnormality of hematology and biochemistry blood panels and were negative for human immunodeficiency virus antibody, p24 antigen and nucleic acids; hepatitis B virus surface antigen and hepatitis C virus antibody. Subjects were excluded if they had any contraindication to BCG vaccination according to the Manufacturer's Data Sheet; had hypersensitivity to any component of the vaccine, severe or multiple allergies; had cardiological, respiratory or neurological disease, a known impairment of immune function or were receiving immunosuppressive therapy; had acute infections; were pregnant or lactating, or capable of becoming pregnant and did not agree to have pregnancy testing before immunization and take effective contraception for the duration of the study; had a problem with substance abuse; had received an investigational agent within 30 days, or been in any other study in the previous 6 months; or were unlikely to complete the study. All subjects provided written informed consent before entering screening. Skin testing with Purified Protein Derivative (PPD, Heaf or Mantoux test) was not performed on subjects to avoid stimulating a circulating T-cell response or gene activation by immune recall.

### 2.2 Oral gut infection challenges

Individual batches of sealed, single dose glass vials containing liquid suspension of 100 mg viable BCG Moreau Rio de Janeiro (approximately 10^7^ viable bacilli) in 5 mL 1.5% sodium glutamate solution were supplied directly by Fundação Ataulpho de Paiva, Brazil, and maintained at 2–8 °C. The same batch was used for each challenge. Volunteers fasted (except water) for a minimum of 2 h before taking a single 100 mg dose in 5 mL, swallowed without additional buffer, on days 0, 28 and 49 (it had originally been proposed to have the third challenge on day 56, but due to an overlap with holidays this was brought forward to day 21 after the second immunization). Volunteers fasted a further 2 h, during which no liquids were allowed in the first 30 min, while volunteers were observed. Apart from oral steroids or other immunosuppressives, there were no restrictions on concomitant medications, which were recorded.

### 2.3 Recording and analysis of symptoms

Following challenge, subjects were issued semi-structured diary cards to record symptoms in an attempt to monitor activation of innate immune system or inflammatory pathways. This elicited symptoms relating to the gastrointestinal and upper respiratory tracts, while allowing free text entry for other symptoms. Subjects graded symptoms as mild, moderate or severe, which were allocated a score of 1, 2 or 3, respectively. To analyze symptoms in association with each challenge, the sum of the symptom severity scores of all symptoms recorded by all subjects on each day in the first 28 days after challenge were summed, to give an aggregate symptom score. The score therefore encapsulates both the frequency and severity of symptoms on any given day for the whole group.

### 2.4 Interferon gamma (IFNγ) responses

Peripheral blood mononuclear cells were separated from heparinised blood by Ficoll discontinuous gradient centrifugation and frozen at −80 °C prior to measurement of frequency of IFNγ-secreting cells and secretion of IFNγ into culture supernatant in response to stimulation with the following antigens: PPD (SSI, Copenhagen) 5 μg/mL, Ag85 peptide pool (LUMC, Leiden) 5 μg/mL or MPB70 (Lionex, Germany) 5 μg/mL; and medium alone or PHA 2 μg/mL, all in AIMV medium (Invitrogen, UK) containing penicillin–streptomycin. Briefly, 1.5 × 10^5^ cells/well were stimulated for 7 days in 96-well plates at 37 °C and 5% CO_2_ in a humidified incubator with antigens or controls, and concentration of supernatant IFNγ measured by ELISA kit (U-CyTech, Netherlands) expressed in pg/mL using a standard on each plate (NIBSC control Human IFNγ rDNA derived, 88/606, NIBSC, UK) and SoftMax software. For ELISPOT, 1 × 10^6^ cells/well (for PHA 3.6 × 10^5^ cells/well) were first stimulated for 18 h in 48-well plates at 37 °C and 5% CO_2_ in a humidified incubator with antigens or controls, and transferred to PVDF-backed 96-well plates (MAHA S45, Millipore, UK) coated with 5 μg/ml anti-human IFNγ mAb 1-D1K (Mabtech, 3420-3-1000) for a further 18 h incubation. Responder cells were detected by sequential incubation with 5 μg/ml anti-human IFNγ mAb biotinylated (Mabtech, 3420-6-250), strepdavidin–alkaline phosphatase (Mabtech, 3310-10), and BCIP/NBT (Sigma, B5655), and spots counted on an automated reader (ViruSpot Elispot reader, AID UK). Values are reported as number of spot forming cells above background number in unstimulated wells, or pg/mL IFNγ in supernatant after subtraction of level in unstimulated wells.

### 2.5 Measurement of gene expression profiles

Subjects returned to the study site at predefined times (Table 1) to have blood drawn. Whole blood was drawn directly into PAXgene Blood RNA System tubes (PreAnalytiX, BD, UK) and RNA extracted according to manufacturer's instructions before freezing at −80 °C. Following QC analysis, samples were selected for amplification and hybridization into Illumina HumanWG-6_V2 arrays from days 0, 2, 4 and 7 after each challenge (see Table 1). Analysis of whole genome gene expression microarrays was performed with GeneSpringGX9 (Agilent Technologies). Samples were normalized using median of all samples baseline transformation and quantile normalization algorithms. Pathway and Gene Ontology (GO) analysis were performed with the novel informatics package InnateDB (www.innatedb.ca). Microarray data has been deposited at ArrayExpress, a MIAME compliant public archive at EMBL-EBI (accession number E-TABM-853).

## 3 Results

### 3.1 Symptoms indicating activation of innate or inflammatory mechanisms

**Fig. 1:**
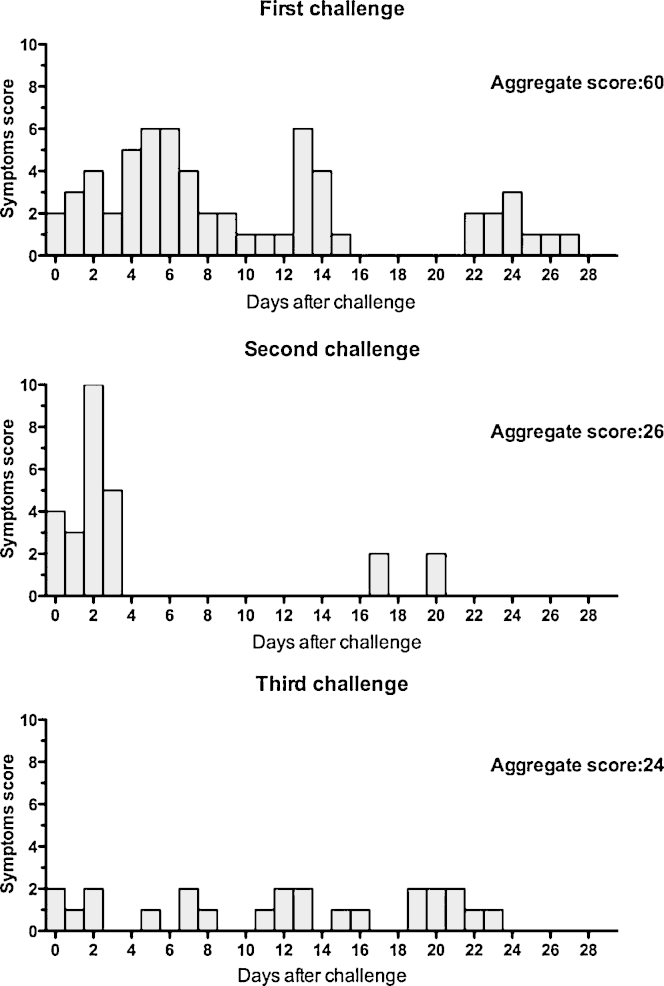
Symptoms after each challenge infection. The symptoms recorded in the diary cards were graded by severity (1, 2 or 3) and a total severity score from all symptoms calculated by adding all the scores recorded by all subjects on each day over the first 28 days after the first and third challenges, and 21 days after the second challenge (as the third challenge was brought forward to 21 days after the second). The aggregate score is the sum of all daily scores after each challenge. Score is higher after the first does, indicating activation of innate immunity, but decreases after following doses.

Seven subjects (5 male and 2 female, ages 22–39, median 27 years) were recruited to receive three sequential oral BCG Moreau Rio de Janeiro (approximately 10^7^ viable bacilli) challenges (see Section 2). All subjects completed all visits. Scoring results of symptoms after each vaccination dose are shown in Fig. 1. One subject reported moderate symptoms (abdominal discomfort and loose stool), and one reported more severe symptoms (loose stools on 2 days). Other symptoms were mild and non-specific. Five subjects reported upper respiratory tract symptoms after the first challenge, none after the second, and one after the third. After each challenge four (different) subjects recorded gastrointestinal symptoms. Interestingly, the frequency and persistence of symptoms was highest after the first challenge (see Fig. 1, total 28-day aggregate score: 60). After the second challenge there were fewer symptoms confined mainly to the first 4 days, with a 28-day aggregate score of 26. After the third challenge there was the lowest number of symptoms, present as a low-level background with an aggregate score of 24.

### 3.2 Adaptive cell mediated immune responses

**Fig. 2:**
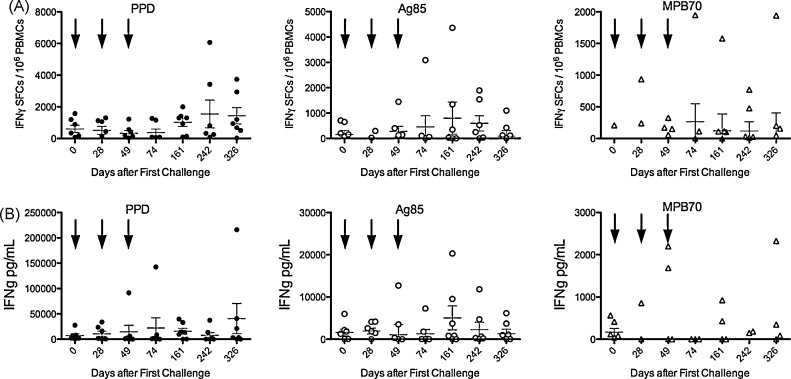
Immune response to challenge infection. Frequency of antigen-specific cell mediated immune responses for individual subjects at various time points after oral challenges (arrows) expressed as (A) IFNγ spot forming cells (SFCs) in response to 36 h *in vitro* stimulation with PPD (closed circles), Ag85 (open circles) or MPB70 (open triangles) measured by ELISPOT assay. Values represent frequency of SFCs in antigen-stimulated wells after subtraction of frequency in unstimulated wells, per 10^6^ PBMCs stimulated. (B) Concentration of IFNγ secreted into culture supernatant after 7-day *in vitro* stimulation with the different antigens, expressed as pg/mL after subtraction of IFNγ concentration in unstimulated wells.

All subjects had received parenteral immunization with BCG in the past, and therefore IFNγ secretion in response to antigen stimulation could be detected at baseline, as expected (Fig. 2). There was little increase in the frequency of cells responding to PPD or Ag85 stimulation detected by ELISPOT until 6 months after the first challenge (3 months after the third—Fig. 2A). This late onset elevated response to PPD persisted until 12 months, whereas that to Ag85 declined from a peak at 6 months, possibly a result of the larger variety of antigens present in PPD.

The detection of IFNγ secretion into supernatant after 7 days *in vitro* stimulation was generally less sensitive than ELISPOT (Fig. 2B), although there was a trend to a response to PPD and Ag85, peaking at 12 and 6 months, respectively, with no response detected to MPB70.

### 3.3 Gene expression analysis using microarrays

Microarray analysis of whole blood from vaccinated individuals showed remarkably limited statistically relevant change in gene expression following each of the vaccine challenges. Out of >48,000 probes, only 6 and 9 genes were significantly differentially expressed at both days 4 and 7, respectively, after the first challenge, compared to day 0 and all these genes were down-regulated (Table 2). Importantly, further challenges did not detectably change gene expression. No pathway or GO term was over-represented on day 4. However, at day 7, an over-representation of GO terms related to cytoskeleton (*p*-value 0.008) and IL6 receptor activity (2 genes, *p*-value 0.01) could be observed. This correlates with pathway analysis, which showed over-representation of IL6 (2 genes, *p*-value 0.0027) signaling and ‘*Vibrio cholerae* and pathogenic *Escherichia coli* (both EPEC and EHEC) infection’ pathways (3 genes, *p*-values 0.017 and 0.016, respectively), as described in InnateDB (www.innatedb.ca) (Table 2). Taken together, these results suggest a lack of significant reactogenicity to the vaccine but enhanced resistance to re-challenge, correlating with the clinical results.

## 4 Discussion

In the present study we were interested in profiling aspects of innate immune activation by repeated oral challenge infection of healthy volunteers with *M. bovis* BCG Moreau Rio de Janeiro vaccine. The oral challenge infections were generally only mildly reactogenic. Scoring of clinical symptoms showed a higher score after the first challenge. Thus, it would appear, based on clinical symptoms, that the first challenge induced the highest acute activation of inflammatory mechanisms, with a shorter burst after the second challenge, and no clinically detectable activity after the third.

The peak PPD response detected (1550 spots/10^6^ PBMCs) was higher than observed previously (450 spots/10^6^ PBMCs at 3 months) after a single oral dose of the same vaccine given in a large volume buffer solution [5]. The higher level of response observed in this study compared to previously published data [5] may reflect a degree of priming by the first two oral challenges, although in this study the ELISPOT assay was different in that an 18-h pre-incubation with antigen was included. No response to MPB70 antigen was detected prior to oral challenge with BCG Moreau, but low-level responses were observed after vaccination. MPB70 is an antigen secreted at high levels by BCG Moreau strain but not the BCG Glaxo strain the subjects probably received in childhood [6]. The lack of high level MPB70 secretion by BCG Glaxo, and thus the lack of immune memory on vaccinated volunteers, probably explains the previous observation that no responses were detectable prior to oral challenge, and when they did occur they were lower than recall responses to Ag85 which is expressed at similar levels by all strains of BCG [7]. Microarray analysis of gene expression correlated with the lack of obvious reactogenicity of the vaccine, only showing a down-regulation of actin and IL6 associated genes.

The BCG oral challenge model was selected as being safe, associated with a mild-moderate degree of reactogenicity in previous studies, and available as an acceptable commercial formulation of attenuated *M. bovis*. A more reactogenic challenge organism (such as partially attenuated strains of *Shigella* or *Salmonella*) may have given more conclusive results, had an acceptable formulation been available. However, we did observe a decline in clinical symptoms with each subsequent oral challenge, suggesting a degree of resistance to challenge was developing. The peak of IFNγ response seen after the third challenge was higher than had been observed in the previous study using only one oral dose [5]. This may imply a degree of priming by the first two challenges and the data suggest that close spacing of oral doses with live BCG may not be optimal to induce an adaptive response, especially one that occurs rapidly. However, we designed the study with the specific aim that the second and third challenges should interfere with the previous ones via the innate and not adaptive immune response. Although there is no direct evidence that subsequent challenges interfered with the immune responses to previous challenge, it remains a possible explanation for the relative lack of response to the second and third challenges. Alternatively, immune responses to mycobacterial infection may take longer to develop, and the close spacing of repeat challenges may not have given sufficient time for an effective memory response to develop before the second and third challenges. As with all studies using cellular readouts in humans, there was considerable within-subject, and between-subject variation, and further larger studies will be needed to confirm the preliminary observations reported here.

In conclusion, although the potential of this approach for monitoring clinical innate immune responses to gut infection via gene activation would appear to be limited, oral challenge infection with BCG Moreau Rio de Janeiro vaccine is safe and immunogenic in healthy volunteers.

## Figures and Tables

**Table 1:** Schedule of visits, samples collected and assays performed.

Visit^a^	2	3	4	5	6	7	8	9	10	11	12	13	15	16	17	18	19	20	21	22	23

Day/month after first challenge	0	2	4	7	14	21	28	30	32	35	42	49	51	53	56	63	70	74	6 months	9 months	12 months

BCG challenge	X						X					X									
PAXGene^b^	x	x	x	x	x	x	x	x	x	x	x	x	x	x	x	x	x	x	x	x	x
Arrays^c^	X	X	X	X			X	X	X	X		X	X	X	X						
CMI^d^	X						X					X						X	X	X	X

a Study visit 14 was omitted.

b Samples of blood taken into PAXGene tubes, RNA extracted and analysis of content and purity performed for these time points.

c Analysis of RNA by Illumina microarray performed for these time points.

d PBMCs separated and *in vitro* culture for IFNγ secretion in response to antigen stimulation performed for these time points.

**Table 2:** Differentially expressed genes after oral BCG vaccination.

Day	Symbol	RefSeq_ID	Fold change	*p*-Value	GO/pathways
4	SNORD13	NR_003041.1	−1.5164351	0.01561	Translational elongation
	LOC654194	XM_942669.1	−1.8388946	0.0283	
	LOC641848	XM_935588.1	−1.5313528	0.03607	
	EEF1B2	NM_021121.3	−1.6000162	0.03859	Translational elongation
	RPL17	NM_001035006.1	−2.1609983	0.04507	Translational elongation
	LOC653773	XM_938755.2	−1.9737101	0.04602	

7	WDR1	NM_017491.3	−1.57219	0.00793	Cytoskeleton/pathogenic *E. coli* infection and *Vibrio cholerae* infection pathways
	JAK1	NM_002227.2	−1.5415655	0.00929	IL6 receptor activity/IL6 signaling pathway
	IL6R	NM_000565.2	−1.5137477	0.01698	IL6 receptor activity/IL6 signaling pathway
	TAGAP	NM_138810.2	−1.6010119	0.01916	Signal transduction
	LOC644063	XR_016547.1	−1.7702552	0.02036	
	ACTG1	NM_001614.2	−1.5455536	0.02069	Cytoskeleton/pathogenic *E. coli* infection and *Vibrio cholerae* infection pathways
	ACTB	NM_001101.2	−1.5035121	0.02776	Cytoskeleton/pathogenic *E. coli* infection and *Vibrio cholerae* infection pathways
	ARHGAP30	NM_001025598.1	−1.5573095	0.04186	Signal transduction
	ALDOA	NM_184041.1	−1.5015596	0.04596	Glucose metabolism
